# 用于治疗非小细胞肺癌的阿法替尼脂质体的制备与包封率的测定

**DOI:** 10.3779/j.issn.1009-3419.2018.09.02

**Published:** 2018-09-20

**Authors:** 晓燕 吕, 君婧 尹, 秀成 杨, 沙 刘, 考祥 孙

**Affiliations:** 264005 烟台，烟台大学新型制剂与生物技术药物研究山 东省高校协同创新中心、分子药理和药物评价教育部重点实验室（烟台大学） School of Pharmacy, Key Laboratory of Molecular Pharmacology and Drug Evaluation (Yantai University), Ministry of Education, Collaborative Innovation Center of Advanced Drug Delivery System and Biotech Drugs in Universities of Shandong, Yantai University, Yantai 264005, China

**Keywords:** 阿法替尼, 脂质体, 硫酸铵梯度法, 紫外分光光度法, 包封率测定, Afatinib, Liposomes, Ammonium sulfate gradient method, Ultraviolet spectrophotometry, Determination of entrapment efficiency

## Abstract

**背景与目的:**

阿法替尼是针对非小细胞肺癌及继发性耐药研发的第二代不可逆表皮生长因子受体抑制剂，目前仅停留在口服给药方式，其生物利用度低，不良反应较多。本研究旨在制备新型药物传递系统-阿法替尼脂质体，并建立包封率的测定方法。

**方法:**

4种不同方法制备阿法替尼脂质体，通过对比包封率和粒径确定最佳制备工艺。

**结果:**

经验证可采用葡聚糖凝胶微柱离心法纯化脂质体，并通过紫外分光光度法测定脂质体的包封率。不同制备方法中硫酸铵梯度法制备的脂质体，包封率约为90.73%，平均粒径为108.6 nm。

**结论:**

硫酸铵梯度法制备阿法替尼脂质体包封率高、粒径小，紫外分光光度法用来测定脂质体的包封率简单易行、准确度高。

## Abstract (en)

**Background and objective:**

Afatinib, a second-generation irreversible epidermal growth factor inhibitor receptor for the development of non-small cell lung cancer and secondary drug resistance, has low bioavailability and adverse reactions due to current oral administration. The aim of this study was to prepare a novel drug delivery system, afatinib liposome, and to establish a method for the determination of encapsulation efficiency.

**Methods:**

Four different preparation methods were used to prepare afatinib liposomes, and the optimal preparation process was determined by comparing the encapsulation efficiency and particle size.

**Results:**

It has been verified that sephadex microcolumn centrifugation can be used to purify afatinib liposomes, and UV spectrophotometry can be employed to determine the entrapment efficiency of liposomes. Among different preparation methods, the encapsulation efficiency of afatinib liposomes prepared by ammonium sulfate gradient method was 90.73% and the average particle size was 108.6 nm.

**Conclusion:**

Ammonium sulfate gradient method can be successfully applied to prepare afatinib liposomes that performed higher encapsulation efficiency and smaller particle size. The UV spectrophotometry employed to determine the liposome encapsulation efficiency was easy operation and with high accuracy.

据2015年中国癌症统计显示，非小细胞肺癌（non-small cell lung cancer, NSCLC）约占肺癌的85%，75%的患者发现时已处于中晚期，转移后5年生存率低至17.1%^[1]^。传统治疗方案一般使用铂类药物进行化疗，但治疗的有效率仅为10%左右。2004年，研究发现，NSCLC与表皮生长因子受体（epidermal growth factor receptor, *EGFR*）突变有关，自此肺癌的治疗进入分子靶向研究阶段。吉非替尼是第一代EGFR酪氨酸激酶抑制剂（tyrosine kinase inhibitor, TKI），对*EGFR*突变的患者疗效优于化疗^[2]^。但是患者在用药一年后出现TKI耐药，研究^[3]^表明这种TKI耐药主要是由于EGFR外显子的苏氨酸-790突变为蛋氨酸（T790M）。第二代不可逆EGFR酪氨酸激酶抑制剂-阿法替尼（afatinib）即是针对TKI继发性耐药开发研制的，它携带一个反应性的丙烯酰胺基，是ATP竞争苯胺基衍生物，能够阻止EGFR、HER2和HER4激酶，形成共价键和不可逆价键。临床研究表明阿法替尼与西妥昔单抗联用对继发性TKI耐药产生明显疗效^[3-5]^。但目前市售阿法替尼为片剂，口服给药生物利用度较低，且不良反应较多，其中胃肠道的不良反应和给药部位异常较为常见，严重者需中断给药或减少剂量进行控制^[6-10]^。为提高患者的生命质量，更好的发挥药效，开发一种具有靶向性、生物利用度高、不良反应少的阿法替尼新型制剂势在必行。

本研究采用脂质体作为药物载体，并在表面链接甲氧基聚乙二醇磷脂（DSPE-PEG2000），实现药物在体内的长循环作用，提高生物利用度，减少药物对胃肠道的刺激，降低不良反应的发生。脂质体的制备方法包括薄膜分散法、逆向蒸发法、有机溶剂注入法、梯度载药法等，其中薄膜分散法、逆向蒸发法和有机溶剂注入法为被动载药，包封率相对较低；而梯度载药法为主动载药，包封率高。本研究先后采用薄膜分散法、逆向蒸发法、改良的乙醇注入法和硫酸铵梯度法制备脂质体，通过葡聚糖凝胶微柱离心纯化脂质体，采用紫外分光光度法测定脂质体的包封率，用Nicomp 380 ZLS激光粒度仪测定脂质体的粒径，进一步优化处方工艺参数，以确定阿法替尼脂质体的最佳制备方法。

## 1 材料与方法

### 1.1 材料

IKA旋转蒸发仪（德国IKA公司），DF-101Z集热式恒温磁力搅拌器（郑州长城科工贸有限公司），电子分析天平（美国Mettler Toledo公司），JY92-Ⅱ超声波细胞粉碎机（宁波新芝生物科技股份有限公司），Nicomp 380 ZLS激光粒度仪（美国PSS公司），离心机Primo R（美国SORVALL公司），UV-2450紫外分光光度仪（日本SHIMADZU公司）。阿法替尼双马来酸盐（afatinib dimaleate）（武汉瑞立升科技发展有限公司，批号：20160615），高纯氢化大豆磷脂（HSPC）（上海艾韦特医药科技有限公司，批号：B50337），胆固醇（CH）（上海缘聚生物科技有限公司），甲氧基聚乙二醇磷脂（DSPE-PEG2000）（烟台芃硕生物科技有限公司，批号：C01126），硫酸铵（天津市致远化学试剂有限公司），氯化钠（天津市恒兴化学试剂制造有限公司），葡聚糖凝胶G-50（华中海威基因科技有限公司），无水乙醇（天津市永大化学试剂有限公司），甲醇（天津市富宇精细化工有限公司）。

### 1.2 方法

#### 1.2.1 制备工艺的选择

##### 1.2.1.1 薄膜分散法

取膜材HSPC、CH、DSPE-PEG2000按质量比4:1:1溶于适量的无水乙醇，旋转蒸发仪减压旋转形成薄膜，加入适量阿法替尼溶液，65 ℃水化20 min，制得脂质体初品。经超声细胞粉碎机200 W 2 min、400 W 6 min后，依次通过0.80 μm、0.45 μm、0.22 μm的微孔滤膜，即得阿法替尼脂质体。

##### 1.2.1.2 逆向蒸发法

取膜材HSPC、CH、DSPE-PEG2000按质量比4:1:1溶于适量的无水乙醇，加入阿法替尼溶液，进行短时超声，直至形成稳定的W/O型乳剂。减压除去乙醇，经超声细胞粉碎机200 W 2 min、400 W 6 min后，依次通过0.80 μm、0.45 μm、0.22 μm的微孔滤膜，即得阿法替尼脂质体。

##### 1.2.1.3 改良的乙醇注入法

取膜材HSPC、CH、DSPE-PEG2000按质量比4:1:1溶于适量的无水乙醇，加入预热至相同温度的阿法替尼溶液，65 ℃水浴搅拌20 min，经超声细胞粉碎机200 W 2 min、400 W 6 min后，依次通过0.80 μm、0.45 μm、0.22 μm的微孔滤膜，即得阿法替尼脂质体。

##### 1.2.1.4 硫酸铵梯度法

取膜材HSPC、CH、DSPE-PEG2000按质量比4:1:1溶于适量的无水乙醇，加入预热至相同温度的200 mmol/L的硫酸铵溶液，65 ℃水浴搅拌20 min，得空白脂质体初品。经超声细胞粉碎机200 W 2 min、400 W 6 min后，依次通过0.80 μm、0.45 μm、0.22 μm的微孔滤膜，得空白脂质体混悬液。将制得的空白脂质体，常温下透析除去脂质体外水相的硫酸铵。以药脂质量比1:8向脂质体混悬液中加入一定体积阿法替尼溶液，60 ℃孵育10 min，即得阿法替尼脂质体。

#### 1.2.2 阿法替尼含量的测定方法

##### 1.2.2.1 最大吸收波长的选择

配制一定浓度的阿法替尼甲醇溶液，取相应量不含阿法替尼的空白脂质体溶于甲醇中，以甲醇作为空白对照溶液，在200 nm-400 nm范围内扫描，确定最大吸收波长。

##### 1.2.2.2 标准溶液的制备

配制浓度为5 μg/mL、7.5 μg/mL、10 μg/mL、15 μg/mL、20 μg/mL、25 μg/mL的阿法替尼甲醇溶液，使用紫外分光光度仪，设定吸收波长343.6 nm，测定吸光度，以吸光度A对浓度C（μg/mL）绘制标准曲线。

##### 1.2.2.3 精密度的考察

配制低、中、高（5 μg/mL、10 μg/mL、20 μg/mL）3种浓度的阿法替尼甲醇溶液，同一天内连续测5次测定吸光度值，计算精密度。

##### 1.2.2.4 重现性的考察

配制10 μg/mL的阿法替尼的甲醇溶液，连续测5次测定吸光度值，计算重现性。

##### 1.2.2.5 方法回收率的考察

配制低、中、高（5 μg/mL、10 μg/mL、20 μg/mL）三个浓度的阿法替尼甲醇溶液，测定吸光度值，并通过标准曲线计算浓度，此浓度与理论浓度相比计算得方法回收率。

#### 1.2.3 脂质体的纯化方法

##### 1.2.3.1 葡聚糖凝胶G-50的预处理

取适量的葡聚糖凝胶G-50置于烧杯中，近沸水浴中加热5 h左右，使凝胶充分溶胀。放凉后用超纯水冲洗3遍，待用。

##### 1.2.3.2 洗脱次数的选择

取脂质体200 μL滴加到葡聚糖微柱的中心，停留2 min后，500 rpm离心5 min，用甲醇定容至5 mL。之后加超纯水500 μL，1, 500 rpm离心5 min，分次进行洗脱，每份洗脱液用甲醇定容至5 mL，于343.6 nm处测定每份洗脱液的吸光度。同法测定阿法替尼溶液的洗脱情况。

##### 1.2.3.3 空白脂质体回收率的考察

取两份空白脂质体200 μL，一份直接用甲醇定容至10 mL，测吸光度为A_0_。一份上样于葡聚糖凝胶微柱，停留2 min后，500 rpm离心5 min。加超纯水500 μL，1, 500 rpm离心5 min，重复洗脱2次。合并洗脱液，用甲醇定容至10 mL，测吸光度为A_1_。通过标准曲线计算C_0_、C_1_，回收率公式为R=（C_1_/ C_0_）×100%。

#### 1.2.4 不同制备工艺脂质体包封率和粒径的测定

对不同工艺制备的脂质体包封率进行测定，用Nicomp 380 ZLS激光粒度仪对脂质体的粒径进行测定，在透射电镜下观察其形态，并对最佳制备工艺所制得的脂质体的载药量进行计算。

载药量（loading efficiency）是指单位重量或单位体积脂质体所负载的药量，LE（%）=We/Wm×100%，式中LE表示脂质体中药物的载药量百分数；We表示包封于脂质体内的药量；Wm表示载药脂质体的总重量。

#### 1.2.5 单因素考察

通过对制备工艺的分析，本研究对HSPC与CH的质量比、afatinib与HSPC的质量比、硫酸铵的浓度、孵育温度与孵育时间等因素进行考察。在其他参数保持不变的条件下，只改变单一因素，以粒径和包封率为指标，考察该因素对脂质体制备工艺的影响。

##### 1.2.5.1 HSPC与CH的质量比对脂质体的影响

在不改变其他因素的条件下，分别考察了HSPC与CH的质量比为2:1、4:1和6:1时对脂质体的包封率和粒径的影响。

##### 1.2.5.2 Afatinib与HSPC的质量比对脂质体的影响

在不改变其他因素的条件下，分别考察了afatinib与HSPC的质量比为1:4、1:8和1:16时对脂质体的包封率和粒径的影响。

##### 1.2.5.3 硫酸铵的浓度对脂质体的影响

在不改变其他因素的条件下，分别考察了硫酸铵的浓度为150 mmol/L、200 mmol/L和250 mmol/L时对脂质体的包封率和粒径的影响。

##### 1.2.5.4 孵育温度对脂质体的影响

在不改变其他因素的条件下，分别考察了孵育温度为50 ℃、60 ℃和70 ℃时对脂质体的包封率和粒径的影响。

##### 1.2.5.5 孵育时间对脂质体的影响

在不改变其他因素的条件下，分别考察了孵育时间为5 min、10 min和15 min时对脂质体的包封率和粒径的影响。

## 2 结果

### 2.1 制备工艺的选择

采用薄膜分散法制得的脂质体，外观较混浊，不易过膜；采用逆向蒸发法制得的脂质体，外观半透明，略带蓝色乳光，较易过膜；采用改良的乙醇注入法制得的脂质体，外观半透明，略带蓝色乳光，较易过膜；采用硫酸铵梯度法制得脂质体，外观半透明，略带蓝色乳光，易过膜。

### 2.2 阿法替尼含量的测定方法

#### 2.2.1 最大吸收波长的选择

阿法替尼溶液在1、2、3处均有吸收（图 1），但因在第2处波长下脂质体膜材的干扰较大，第3处已接近近紫外端，故选择第1处——343.6 nm为最大吸收波长。

**1:**
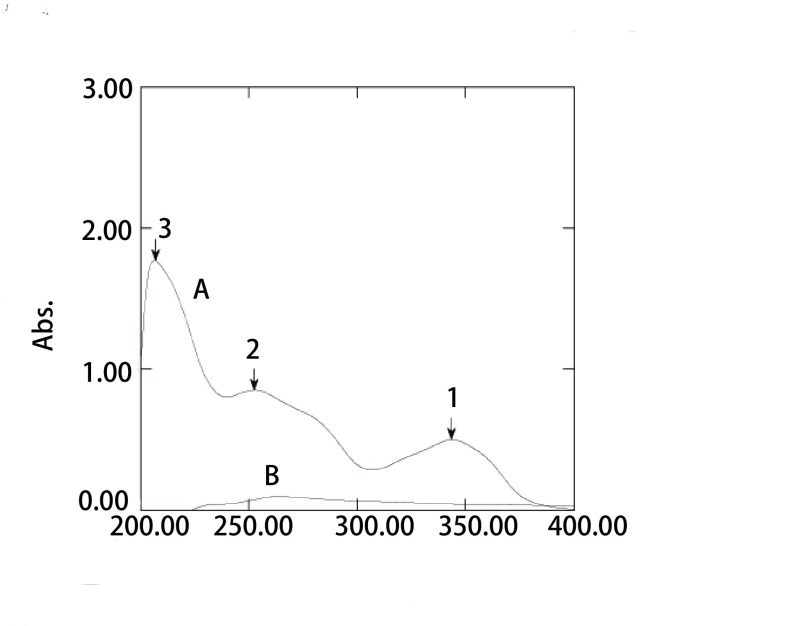
紫外最大吸收波长扫描图。A：阿法替尼；B：空白脂质体。 The maximum absorption wavelength scanning of ultraviolet light. A: Afatinib; B: Blank liposomes.

#### 2.2.2 标准溶液的制备

计算吸光度A对浓度C的回归方程为y=0.027, 2x+0.003, 6，R^2^=0.999, 4（图 2），结果表明，在浓度范围为5 μg/mL-25 μg/mL时，标准曲线的线性良好。

**2:**
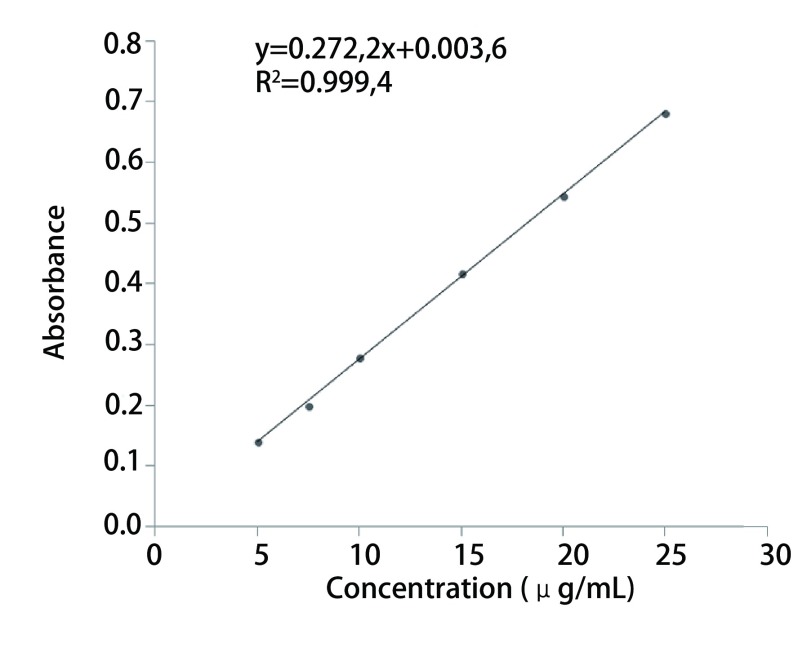
阿法替尼溶液标准曲线 Standard curve of afatinib solution

#### 2.2.3 精密度的考察

经测定，阿法替尼溶液低、中、高三种浓度的RSD值均小于1%（表 1），结果表明方法的精密度良好。

**1:** 阿法替尼溶液精密度测定结果（*n*=5） The precision results of afatinib solution (*n*=5)

Concentration (*μ*g/mL)	Found concentration (*μ*g/mL)					Mean concentration (*μ*g/mL)	RSD (%)
5.00	5.06	5.07	5.06	5.07	5.07	5.07	0.13
10.00	10.15	10.17	10.16	10.18	10.18	10.17	0.11
20.00	19.97	19.97	19.97	19.95	19.96	19.96	0.05

#### 2.2.4 重现性的考察

连续测定5次，阿法替尼溶液（10 μg/mL）的RSD值均小于1%（表 2），结果表明方法的重现性良好。

**2:** 阿法替尼溶液重现性测定结果（*n*=5） The reproducibility results of afatinib solution (*n*=5)

Number	Absorbance	Found concentration (*μ*g/mL)	Mean concentration (*μ*g/mL)
1	0.280, 29	10.17	10.18
2	0.279, 85	10.16	
3	0.280, 90	10.19	
4	0.280, 36	10.18	
5	0.280, 59	10.18	

#### 2.2.5 方法回收率的考察

经测定，方法的回收率在99.74%-101.68%之间（表 3），结果表明方法的回收率良好。

**3:** 阿法替尼溶液方法回收率结果（*n*=5） Theresults of recovery experiments of afatinib solution (*n*=5)

Concentration (*μ*g/mL)	Found concentration					Recovery rate (%)	RSD (%)
5.00	5.05	5.05	5.05	5.05	5.06	101.08	0.02
10.00	10.17	10.18	10.16	10.17	10.16	101.68	0.05
20.00	19.94	19.92	19.94	19.93	20.01	99.74	0.17

### 2.3 脂质体的纯化方法

#### 2.3.1 洗脱次数的选择

实验结果表明，用超纯水洗脱两次时脂质体已洗脱99.17%（表 4），而此时阿法替尼溶液不被洗脱，因此选择洗脱两次纯化脂质体。

**4:** 不同洗次数对脂质体和阿法替尼溶液洗脱结果影响（*n*=3） Effect of different elution times on the results of liposomes and afatinib solution (*n*=3)

Elution times	Liposomes absorbance	Solutions absorbance
1	0.356, 35±0.000, 11	0.004, 43±0.000, 24
2	0.048, 23±0.000, 27	0.003, 31±0.000, 36
3	0.004, 13±0.000, 31	0.002, 00±0.000, 12
4	0.002, 56±0.000, 28	0.002, 93±0.000, 23
5	0.000, 55±0.000, 25	0.000, 57±0.000, 15
6	0.000, 31±0.000, 07	0.000, 08±0.000, 01

#### 2.3.2 空白脂质体回收率的考察

经计算，空白脂质体的回收率R为99.3%，表明葡聚糖凝胶微柱在此洗脱条件下对空白脂质体几乎没有吸附。

### 2.4 包封率方法的确立

取脂质体200 μL滴加到葡聚糖微柱中心，停留2 min后，500 rpm离心5 min。随后加超纯水500 μL，1, 500 rpm离心5 min，重复洗脱2次。合并洗脱液，用甲醇定容至10 mL，测吸光度为A_1_。取脂质体200 μL，直接用甲醇定容至10 mL，测吸光度为A_0_。通过标准曲线计算C_1_、C_0_，包封率根据公式EE=（C_1_/C_0_）×100%计算。

### 2.5 不同制备工艺脂质体包封率和粒径的测定

采用建立的包封率测定方法对四种不同制备工艺的脂质体进行测定，结果表明硫酸铵梯度法的包封率最高，为90.73%（表 5），平均粒径为108.6 nm（图 3）。经透射电镜观测，脂质体呈球形，形态规则完整，可观察到双层膜结构，粒径分布均匀（图 4），因此选择硫酸铵梯度法来制备阿法替尼脂质体。计算用硫酸铵梯度法制备的脂质体的平均载药量为7.52%。

**5:** 不同工艺对脂质体包封率和粒径影响（*n*=3） Effect of different process on encapsulation efficiency and particle size (*n*=3)

Preparation process	Encapsulation efficiency (%)	Particle size (nm)
Film-dispersion method	55.21±2.68	302.3±0.41
Reverse evaporation method	35.62±3.21	202.8±0.38
Improved ethanol injection method	14.15±1.31	234.0±0.31
Ammonium sulfate gradient method	90.73±1.89	108.6±0.28

**3:**
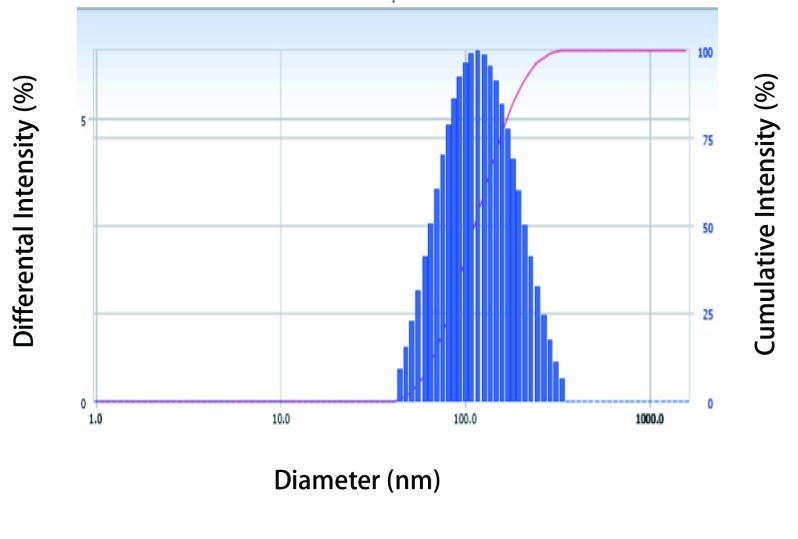
硫酸铵梯度法制备脂质体粒径分布（粒径：108.6 nm，P.I.：0.154） Particle size distribution of liposomes prepared by ammonium sulfate gradient method (particle size: 108.6 nm, P.I.: 0.154)

**4:**
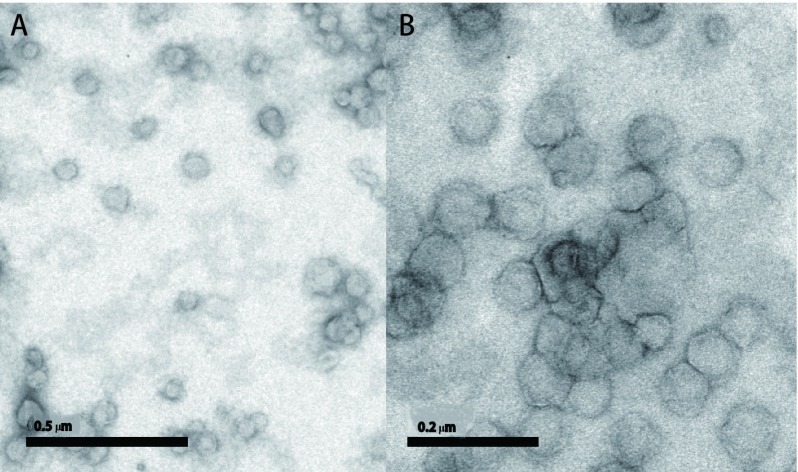
硫酸铵梯度法制备脂质体透射电镜照片。A：0.5 *μ*m；B：0.2 *μ*m。 Transmission electron micrograph of liposomes prepared using ammonium sulfate gradient method. A: 0.5 *μ*m; B: 0.2 *μ*m.

### 2.6 单因素考察

当HSPC与CH的比例为4:1（表 6），afatinib与HSPC的比例为1:8（表 7），硫酸铵的浓度为250 mmol/L（表 8），孵育温度为60 ℃（表 9），孵育时间为10 min（表 10）时，脂质体的包封率高，粒径小。因此，最终确定脂质体的制备工艺为：取膜材HSPC、CH、DSPE-PEG2000按质量比4:1:1溶于适量的无水乙醇中，于65 ℃水浴中加热溶解，加入预热至相同温度的250 mmol/L的硫酸铵溶液，65 ℃水浴搅拌20 min，得空白脂质体初品。经超声细胞粉碎机200 W 2 min、400 W 6 min后，依次通过0.80 μm、0.45 μm、0.22 μm的微孔滤膜，得空白脂质体混悬液。将制得的空白脂质体常温下透析，除去脂质体外水相的硫酸铵。以药脂比1:8向此梯度脂质体混悬液中加入一定体积的阿法替尼溶液，60 ℃孵育10 min。即得阿法替尼脂质体。

**6:** HSPC与CH比例对脂质体包封率和粒径的影响（*n*=3） Effect of the ratio of HSPC and CH on liposome encapsulation efficiency and particle size (*n*=3)

HSPC: CH (w/w)	Encapsulation efficiency (%)	Particle size (nm)
2:1	79.79±2.21	125.7±0.28
4:1	88.86±1.18	125.9±0.71
6:1	87.12±1.58	105.9±0.53

**7:** Afatinib与HSPC比例对脂质体包封率和粒径的影响（*n*=3） Effect of the ratio of afatinib to HSPC on liposome encapsulation efficiency and particle size (*n*=3)

Afatinib: HSPC (w/w)	Encapsulation efficiency (%)	Particle size (nm)
1:4	87.80±2.80	141.4±0.18
1:8	91.6±1.34	129.7±0.25
1:16	28.5±3.22	142.3±0.83

**8:** 硫酸铵浓度对脂质体包封率和粒径的影响（*n*=3） Effect of ammonium sulfate concentration on liposomes encapsulation efficiency and particle size (*n*=3)

Concentration (mmol/L)	Encapsulation efficiency (%)	Particle size (nm)
150	88.40±4.22	117.0±0.67
200	94.33±2.42	103.1±0.23
250	97.70±3.12	109.7±0.15

**9:** 孵育温度对脂质体包封率和粒径的影响（*n*=3） Effect of incubation temperature on liposome encapsulation efficiency and particle size (*n*=3)

Incubation temperature (℃)	Encapsulation efficiency (%)	Particle size (nm)
50	64.93±1.74	127.6±0.21
60	90.09±3.64	116.5±0.09
70	89.13±2.57	133.5±0.16

**10:** 孵育时间对脂质体包封率和粒径的影响（*n*=3） Effect of incubation time on liposome encapsulation efficiency and particle size (*n*=3)

Incubation time (min)	Encapsulation efficiency (%)	Particle size (nm)
5	64.74±3.12	123.3±0.21
10	90.13±1.55	122.7±0.33
15	54.32±2.90	127.2±0.17

## 3 讨论

脂质体的制备方法包括薄膜分散法、逆向蒸发法、有机溶剂注入法、梯度载药法等，其中薄膜分散法多用于脂溶性药物的包载，其成膜的均匀性较为关键，所制得的脂质体粒径较大；逆向蒸发法适于包封水溶性的药物，制得的脂质体内水相较大且包封率较高；有机溶剂注入法操作简单，尤其是乙醇注入法可以避免高毒性的有机溶剂残留问题，在近年的放大生产研究中较为常用；而梯度载药法为主动载药的制备方法，适用于水溶性药物的包载，制得的脂质体包封率高。本研究先后采用薄膜分散法、逆向蒸发法、改良的乙醇注入法和硫酸铵梯度法来制备脂质体，通过对比4种制备方法的包封率和粒径，最终确定制备阿法替尼脂质体的最优工艺为硫酸铵梯度法。硫酸铵梯度法属于梯度载药法的一种，其方法是先制备空白脂质体，在空白脂质体内外形成离子梯度，水溶性的阿法替尼双马来酸盐在离子梯度的作用下进入空白脂质体，并在脂质体内与硫酸铵形成溶解度较小的盐，使药物滞留在脂质体内，此方法制得的脂质体包封率高，粒径小。

常用的脂质体纯化的方法有柱过滤法、超滤法、离心法等。本研究先后考察葡聚糖凝胶过滤法、超滤法、阳离子交换树脂法和葡聚糖微柱离心法。葡聚糖凝胶过滤法虽然可以达到分离的效果，但是操作繁琐，耗时较长，不易操作；超滤法所用的超滤管的膜材对阿法替尼有吸附作用，不能有效地纯化脂质体，导致包封率测定不准确；阳离子交换树脂法，操作简单，因阿法替尼的理化性质，游离药物可完全吸附于微柱上，但是阳离子交换树脂为黄色，虽预处理时已洗净至肉眼观察为无色，但其对测定仍有干扰，所测包封率偏高；而葡聚糖微柱离心法，操作简单，能有效地纯化脂质体，经验证后选定为脂质体纯化的方法。

常用的测定药物含量的方法有：紫外分光光度法和高效液相色谱法等。高效液相色谱法测定准确，但是操作繁琐，进样前需再次过滤样品，并需充分平衡色谱柱；紫外分光光度法操作简便，测样时间短，经验证可满足样品测定所需条件，故选用紫外分光光度法作为测定包封率的方法。

本研究采用硫酸铵梯度法成功制备阿法替尼脂质体，并通过葡聚糖微柱离心纯化脂质体，利用紫外分光光度法测定包封率，操作简单，可重复性好。本研究为阿法替尼的给药方式提供了新的思路，在减轻患者的不良反应方面做出了努力，为阿法替尼的广泛应用打下了基础。
